# The Hour of Delivery

**Published:** 1888-09

**Authors:** J. G. Swayne


SOME persons may be disposed to think that those who attempt to determine by statistics at what hour delivery most frequently occurs, are taking a great deal of trouble about a question which is more curious than useful. But there are others who consider that no investigation can be utterly useless which contributes the smallest amount of information respecting any of the laws or operations of Nature. I, myself, belong to this latter class, and consequently have taken the trouble to ascertain how far my own experience confirms that of another observer whose conclusions were published in the *British Medical Journal* for January 7th, 1888. I quote *verbatim* the article which contained them :

"THE HOUR OF DELIVERY.

"An industrious statistician has recently contributed to a medical journal at Lille the results of an analysis of 1,000 labours, with the precise hour at which delivery took place. He reports that 45 per cent. of the labours terminated between 8 a.m. and 8 p.m., and 56 per cent.

between 8 p.m. and 8 a.m. Pushing the inquiry a step further, he found that 23 per cent. terminated between 6 a.m. and mid-day; 22 per cent. between mid-day and 6 p.m.; 27 per cent. between 6 p.m. and midnight; and 54.1 per cent. during the night—that is to say, between midnight and 6 a.m. These figures only apply to normal labours, in which nothing interfered with the physiological course; so that they go far to confirm the popular notion which admits the greatest frequency of delivery at night, 'conception generally taking place at about that time.'"

In order to test these conclusions I referred to my own Register of Obstetric Cases, which I have kept ever since I began practice, and in which I have noted the exact hour of delivery in every case. Like the observer above mentioned, I carefully excluded all cases in which there was any interference whatever with the natural process, either by medicines, by the hand, or by instruments. I therefore limited my observations to head-presentations, and to those only in which the vertex presented with the occiput in the anterior semicircle of the pelvis, because in all others manual interference is nearly always necessary. For the same reason, breech, knee, foot, and arm-presentations are excluded; and, in short, all labours in which for any cause it was necessary to expedite delivery by ergot of rye, by the hand, or by instruments. By taking these precautions, and dating back from the beginning of the present year, I was enabled, in a period of about thirty years, to obtain statistics of 1,000 perfectly normal labours.

I will now compare my own figures with those just quoted from the *British Medical Journal*. These latter show that 45 per cent. of the labours terminated between 8 a.m. and 8 p.m., and 56 per cent. between 8 p.m. and

8 a.m. My own figures yield a per-centage of 45.1 between 8 a.m. and 8 p.m., and 54.9 between 8 p.m. and 8 a.m., thus showing that the conclusions to be drawn from each set of cases are very nearly identical. From some unaccountable error, the 56 per cent. in the first set of figures is one too many, for 45 + 56 = 101. If, therefore, I subtract this 1, the numbers per cent. will be 45 and 55; and if from my own figures, 45.1 and 54.9, I subtract the one-tenth from the first and add it to the nine-tenths of the second, I shall get the numbers 45 and 55 per cent. respectively, just as in the first set of figures. With respect to my own 1,000 cases, I find that more conclusive results are arrived at by dividing the 24 hours into three periods of eight hours each, than into four of six hours.

For instance, I find that in the first eight hours, viz., from 1 a.m. to 9 a.m., there were 424 deliveries out of 1,000; in the next eight hours, from 9 a.m to 5 p.m., there were 289; and in the last, from 5 p.m. to 1 a.m., 287. Thus it will be observed that about three-sevenths of the 1,000 occurred in the first period of eight hours, and only about two-sevenths in each of the other periods —the numbers in each being very nearly equal.

The annexed table (No. I.) shows the number of deliveries which took place in each hour; but I need not take up time in enumerating these: suffice it to say that the greatest number per hour took place between 4 and 5 a.m., and was as many as 63; the next greatest number, 60, was between 3 and 4 a.m.; none of the other numbers reached 60. On the contrary, the smallest number per hour was 27, and occurred between 2 and 3 p.m.; and this was the only hour in which the number fell below 30.

In the 1,000 labours from which my statistics are derived, there are only 163 first labours, which is scarcely one-sixth of the whole number, and much less than the usual proportion; for in ordinary midwifery practice, first labours are at least one-fifth, or even one-fourth, of the whole number attended. This difference, however, is accounted for by the circumstance that by far the majority of forceps cases are first labours; and instrumental labours are excluded from this list.

No. I.

THE HOUR OF DELIVERY IN 1,000 LABOURS.

| | | | |
|---|---|---|---|
| From 1 to 2 a.m. | 40 | births | 424 |
| ,, 2 to 3 ,, | 53 | ,, | |
| ,, 3 to 4 ,, | 60 | ,, | |
| ,, 4 to 5 ,, | 63 | ,, | |
| ,, 5 to 6 ,, | 51 | ,, | |
| ,, 6 to 7 ,, | 49 | ,, | |
| ,, 7 to 8 ,, | 53 | ,, | |
| ,, 8 to 9 ,, | 55 | ,, | |
| ,, 9 to 10 ,, | 32 | ,, | 289 |
| ,, 10 to 11 ,, | 50 | ,, | |
| ,, 11 to 12 ,, | 30 | ,, | |
| ,, 12 to 1 p.m. | 36 | ,, | |
| ,, 1 to 2 ,, | 38 | ,, | |
| ,, 2 to 3 ,, | 27 | ,, | |
| ,, 3 to 4 ,, | 38 | ,, | |
| ,, 4 to 5 ,, | 38 | ,, | |
| ,, 5 to 6 ,, | 30 | ,, | 287 |
| ,, 6 to 7 ,, | 36 | ,, | |
| ,, 7 to 8 ,, | 41 | ,, | |
| ,, 8 to 9 ,, | 37 | ,, | |
| ,, 9 to 10 ,, | 44 | ,, | |
| ,, 10 to 11 ,, | 31 | ,, | |
| ,, 11 to 12 ,, | 34 | ,, | |
| ,, 12 to 1 a.m. | 34 | ,, | |
| | 1,000 | | 1,000 |

No. II.

163 PRIMIPAROUS LABOURS. HOUR OF DELIVERY.

| | | | | |
|---|---|---|---|---|
| From 1 to 2 a.m. | ... ... ... | 5 | births | 60 |
| ,, 2 to 3 ,, | ... ... ... | 5 | ,, | |
| ,, 3 to 4 ,, | ... ... ... | 10 | ,, | |
| ,, 4 to 5 ,, | ... ... ... | 12 | ,, | |
| ,, 5 to 6 ,, | ... ... ... | 7 | ,, | |
| ,, 6 to 7 ,, | ... ... ... | 9 | ,, | |
| ,, 7 to 8 ,, | ... ... ... | 8 | ,, | |
| ,, 8 to 9 ,, | ... ... ... | 4 | ,, | |
| ,, 9 to 10 ,, | ... ... ... | 6 | ,, | 57 |
| ,, 10 to 11 ,, | ... ... ... | 11 | ,, | |
| ,, 11 to 12 ,, | ... ... ... | 5 | ,, | |
| ,, 12 to 1 p.m. | ... ... ... | 11 | ,, | |
| ,, 1 to 2 ,, | ... ... ... | 4 | ,, | |
| ,, 2 to 3 ,, | ... ... ... | 7 | ,, | |
| ,, 3 to 4 ,, | ... ... ... | 6 | ,, | |
| ,, 4 to 5 ,, | ... ... ... | 7 | ,, | |
| ,, 5 to 6 ,, | ... ... ... | 5 | ,, | 46 |
| ,, 6 to 7 ,, | ... ... ... | 6 | ,, | |
| ,, 7 to 8 ,, | ... ... ... | 6 | ,, | |
| ,, 8 to 9 ,, | ... ... ... | 8 | ,, | |
| ,, 9 to 10 ,, | ... ... ... | 11 | ,, | |
| ,, 10 to 11 ,, | ... ... ... | 3 | ,, | |
| ,, 11 to 12 ,, | ... ... ... | 4 | ,, | |
| ,, 12 to 1 a.m. | ... ... ... | 3 | ,, | |
| | | 163 | | 163 |

The number of first labours is too small to enable one to draw any definite conclusions from them as to the hour of delivery; but it will be seen from the annexed table (No. II.) that on the whole they tend to confirm the results arrived at from the statistics of the 1,000 cases. Thus in the first 12 hours, reckoning from 1 a.m. to 1 p.m., there were 93 deliveries, and in the second 12 hours, from 1 p.m. to 1 a.m., there were 70 only. In the first period of eight hours, beginning at 1 a.m., there were 60 deliveries; in the second 57, and in the third 46. It will be remembered that in the 1,000 cases there were 424 in the first period of eight hours, 289 in the second, and 287 in the third. It will thus be seen that in the 163 first labours, the first period of eight hours is the most prolific, and the two last least so, just as in the 1,000, but not to so marked a degree. In the 1,000 cases there is a great preponderance of numbers in the first eight hours, and a great decrease in the second and third periods; the numbers in these two last being nearly equal. In the 163 first labours, as in the 1,000, the greatest number of deliveries per hour (12) takes place between 4 and 5 a.m.; but the preponderance during the first eight hours is not so marked; nor is there any very marked diminution until we come to the third period, when the numbers fall from 57 to 46. This discrepancy, no doubt, is occasioned by the prolongation of the last stage of the labour in primiparæ, owing to the resistance afforded by the perineum and vaginal orifice; the delivery being sometimes postponed for hours from this cause.

There is one curious coincidence between the statistics of my 1,000 cases and the 163 first labours, and that is, that in both the three consecutive hours which have the smallest number of deliveries are from 10 p.m. to 1 a.m. In the first the number is 99, whereas in all the other periods of three hours it is above 100; and in the second it is only 10, which is far below the number in any other period of three hours. It is difficult to assign any plausible reason why in my 1,000 cases the number of deliveries should have been lowest in the three hours just mentioned, and then in the next three hours should have suddenly risen from 99 to 153, and should have reached the acme 163 in the three hours following these; whilst in the primiparæ the numbers in each of these periods of three hours are respectively 10, 20, and 28, the first being the lowest and the last the highest recorded in any period of three hours. Perhaps, however, this is a mere coincidence peculiar to the 1,000 cases observed by me. The same, however, cannot be said regarding the fact upon which there is such a marked agreement between the Lille observer and myself; viz., as to the great predominance of labours in the eight hours immediately following midnight. This is far too marked and evident to be the result of a mere coincidence, and leads me to allude briefly to the theory that this result is owing to the time when conception usually takes place. To make this theory at all plausible, we require a great number of observations, not only as to the time of conception and delivery in man, but in the lower animals, especially the mammalia.

Owing to ideas of decency and propriety, the sexual act in man is usually performed during the night; but, with the exception, perhaps, of the cat tribe, there is no such marked preference for nocturnal copulation in the lower animals; and in some domesticated animals, especially in horses and cattle, the hour of copulation is regulated by the convenience of men, whose business it is to bring the males and females together; and this usually happens in the daytime. Again, if there is any kind of coincidence between the time when conception and when delivery takes place in man, the greatest number of deliveries ought to happen between the hours of 10 p.m. and 1 a.m., which is just the time when, as I have observed above, we have the smallest number. If, however, we suppose that the period of conception is the same as that in which labour begins, when the os uteri begins to open, and not that in which the labour ends, we should get a very probable solution of the difficulty, provided the greater number of labours lasted only five or six hours: but we know, on the contrary, that the average number last at least double this time; and so, if this theory were correct, the greatest number of deliveries ought to happen in the afternoon, which we know is directly contrary to the facts observed. On the other hand, there is, I think, every reason to believe that, in most cases, the process of delivery first begins in the daytime.

Every man who has had much experience in midwifery knows how very common it is, just before he goes to bed, for him to get an unpleasant reminder in the shape of a message from a parturient woman to say that his services will probably be required some time during the night. He knows also how very often his patient's anticipations are correct; and, as our statistics abundantly prove, that the process of childbirth has a peculiar tendency to terminate in the small hours of the night. I have shown that at 1 a.m. the number of deliveries per hour suddenly rises from 34 to 40, and gradually increases until it attains its acme of 63 between 4 and 5 a.m., and then gradually declines to 55 between 8 and 9 a.m., when there is a sudden drop to 32 between 9 and 10 a.m., as soon as the hours are represented by double figures. It has often been said that death usually occurs in the small hours of the night; if so, there is a singular coincidence between the hour of entrance into the world and exit from it. Is there any analogy between the relaxation of the sphincters in the first case, and the same process in the second, especially with regard to the circular fibres of the os uteri? However this may be, there is no doubt, I think, that the popular notion, that most deliveries take place in the night, is well founded; and therefore he who is much engaged in midwifery practice must make up his mind to sacrifice many hours of much-needed rest. In another way he is as hard-worked as Longfellow's *Village Blacksmith;* but, like him, he cannot say:

> "Each morning sees some task begun,
> Each evening sees it close;
> Something attempted, something done,
> Has earned a night's repose."

